# 

**Published:** 1920-01

**Authors:** 


### SUBJECT INDEX TO VOLUME LXXIV

EVENT AND COMMENT

Adenoids and Tonsils as Related to Teeth, 501.

Bogus American Dental Diplomas in England, 3.

Criticism of Dental Hygienists, 103.

Dental Cosmos Celebrates a Birthday, 52.

Dental Prophylaxis and Hygiene, 401.

Forsythe Infirmay Report, 204.

Government Tax on Dental Supplies, 202.

Improvidence of Dentists, 252.

Index of Dental Literature, 554.

National Dental Association, 451.

Nineteen-Twenty, 1.

Pain and Preventive Dentistry, 352.

Passing of the Faithful, 52.

Proposed Excise Tax on Gold, 202.

The Bridge Problem, 101.

The Necessity for Balance in Practice, 452.

Tonsil-Adenoid Clinic, 503.

University of Rochester, 301.

What's the Matter With Dentistry? 551.

FORMAL PAPERS

Advantages of Intensive Dental Courses. By J. M. Hale, 227.

Amalgam Filling Technic. By William E. Harper, 13.

A New Species of Dentist. By Thomas J. Barrett, 104.

Army Medical Corps, 185.

Articulating Artificial Dentures. By O. A. Weiss, 267.

Asepsis in Root Canal Operations. By Elmer S. Best, 174.

A Theory as to the Etiology of Pyorrhea. By Stanley Colyer, M.D., 526.

Blood Condition A Controlling Factor in Extractions. By Thos. B. Hartzell, 24.

Blood Pressure in Surgical Prognosis. By A. H. Miller, M.D., 120.

Casting Clasp and Saddles in One Piece. By F. E. Roach, 20.

Clinical Study of End Results of Some Focal Infections. By Bruce W. Fontain, 303.

Crown and Bridge-Work. By F. W. Frahm, 307, 411.

Combination Impressions for Accurate Fitting Dentures. By Victor H. Sears, 153.

Complement Fixation Tests as Applied to Dental Practice. By Angelo Zabriskie, 283.

Compulsory Health Insurance. By Jerry McQuade, 435.

Conservation of the Dental Pulp. By Herman Prinz.

Consideration for Others. By C. N. Johnson, 519.

Correction of Malocclusion in Artificial Dentures. By M. M. House, 403.

Council on Pharmacy and Chemistry, Present and Future. By W. A. Puckner, 256.

Dangers Incurred by the Radiographer. By Charles A. Eller, 133.

Dental Hygiene for Children. By G. Walter Dittmar, 480.

Dental Hygienists in Orthodontia Practice. By H. B. Hamilton, 573.

Dental Scientific Research, 422.

Dental Surgery and Organic Heart Disease. By P. J. Calvey, M.D., 383.

Dentistry and the Gold Excise, 379.

Double Grip Cast Clasps for Bridges. By F. E. Roach, 564.

Eckerman on Osmosis and Caries. By Wm. Rushton, 74.

Enlarged Thyroid Due to Impacted Molar. By A. B. French, M.D., 182.

Epicoectomy and Perfect Root Canal Operations. By S. Sidney Gross, 68.

Ethical Problems. By G. W. Clapp, 425.

Focal Infection. By Russell W. Bunting, 416.

Focal Infection, Its Prevalence and Control. By M. Rattner, 207.

Germ Diseases and Methods of Control. By C. E. A. Winslow, M.D., 322.

Gold Foil in Anterior Bridges. By F. H. Entrinken, 392.

Honest Dentistry. By M. A. Fink, 436.

Holidays and National Vigor, 183.

How to Secure X-Ray Protection. By H. T. Fischer, 237.

How to Treat Periodontoclasia. By C. M. Gearhart, 361.

Hypnotic Suggestion in Dental Practice. By Betto Toplan, 82.

*In re* Itinerant Dental Instructors, 274.

Medical Phase of Tonsil-Adenoid Clinic. By Albert D. Kaiser, M.D., 517.

Memorial Resolution, Drs. B. Holly Smith and Henry W. Morgan, 135.

Nerve-End Cells in the Dental Pulp. By J. Howard Mummery, 386.

Physiology of Oral Hygiene. By J. Sim Wallace, 555.

Prevention of Disease. By a Physician, 276.

Problem of Pyorrhea Alveolaris. By Stanley Colyer, M.D., 578.

Professionalism versus Commercialism. By C. N. Johnson, 472.

Pulpless Teeth, Shall They Be Extracted? By Thomas Cowling, 522.

Pulps and Pulpless Teeth. By Elmer S. Best, 317.

Radicalism. By F. C. Brush, 126.

Rationalism in Impression Work. By F. W. Frahm, 4.

Regeneration in Chronic Dento-Alveolar Abscess. By Julio Endelman, 373.

Relation Between General Digestive Conditions and Mouth Conditions. By M. Marion Null, M.D., 222.

Removable and Fixed Bridge-Work. By F. W. Frahm, 53.

Roach versus Bonwill Clasps. By H. W. Allwine, 32.

Root Canal Filling and Health. By Edgar D. Coolidge, 164.

Root Canal Operations. By Elmer S. Best, 271.

Scientific Data on the Grinding and Burring of Teeth. By Emil Huet, 487.

Standardizing Vascular Depression. By E. J. McKesson, M.D., 122.

Surgical Phase of the Tonsil-Adenoid Clinic. By Edwin S. Ingersol, M.D., 514.

Systemic Management of Periodontoclasia. By Elmer S. Best, 363.

Teeth and the Worker. By James Burnett, 365.

The Dental and Adenoid Clinics. By Harvey J. Burkhart, 509.

The Dentist at Thirty-five. By G. W. Clapp, 426.

The Interrelations of Orthodontic Malformations with Diseases of the Nose and Throat. By G. C. Dittman, M.D., 78.

The Necessity of a Library. By D. M. Cattell, 468.

The Sixth Year Molar. By John E. Gurley, 59.

The Translucent Filling Cement. By F. N. Doubleday, 454.

Tonsils, Adenoids, and the Teeth. By George W. Goles, M.D., 512.

Tooth Brush and Methods of Cleaning Teeth, 353.

Trench Mouth. By Guy A. Karr, 281.

What Are the Chances You Will Die Rich? By R. D. Wycoff. 253.

INDENTS

Acid Producing Bacteria and Dental Caries, 87.

Alcohol a Stimulant, 91.

A Lingual Matrix. By G. S. Junkerman, 88.

American Institute of Dental Teachers and Officers, 196.

An Aid for Contour Clasps. By L. M. Baughman, 397.

A New Price for Drugs, 342.

Anesthetists, 343.

Anesthetizing and Sterilizing the Gum Locally. By F. J. Crymes, 291.

Announcements of May and June Dental Meetings, 196.

An Iodine Lotion. By N. Rudin, 291.

Another Case of Focal Infection, 444.

Are Non-Vital Teeth Foreign Bodies? By R. Ottolengui, 442.

Bacteria in Tooth Pulps, 441.

Bridge-Work and Mobility of the Teeth. By Dr. Hovestadt, 93.

British Dental Association, 492.

Build Now Movement, 295.

Caries,—Eckerman, 74.

Caries and Mouth Breathing. By W. H. Brown, 123.

Cast Clasps, 539.

Casting a Richmond with Steele's Facing. By Joseph Homer, 341.

Cementing a Crown to a Bridge, 43.

Cement Destroyed by Water Contact, 195.

Clasps. By Edward Kennedy, 143.

Clean Food, 38.

Commercial versus Surgical and Remedial Appliances, 494.

Conforming the Wax for Denture Casting. By F. W. Frahm, 292.

Consultations Among Dentists, 191.

Curettage, 536.

Dental Abscesses and Toxemia of Pregnancy, 42.

Dental Cysts, 395.

Dental Education, 37.

Dental Health and Education in New York State. By W. H. Leak, 146.

Dental Health. By Rea Proctor, 447.

Dental Hygienists Graduate, 249.

Dental Hygiene. By C. N. Johnson, 490.

Dental Pain. By Edmund Kells, 243.

Devitalization of Pulps in Bridge-Work. By Dr. Hovestadt, 94.

Dichloramin T Solution. By H. Prinz, 193.

Dignity of Dental Service. By C. N. Johnson, 395.

Dietetic Value of Nuts, 197.

Duplicating Plaster Casts, 291.

Eating to Live, 537.

Entering Dental Practice. By O. R. Ottolengui, 241.

Exclusion of Moisture and Asepsis, 42.

Extension for Prevention. By J. V. Conzett, 496.

Extracting Upper Canines, 496.

Fainting Under Novocaine, 533.

Focal Infection and the Eyes. By W. R. Ackland, 96.

Functional Articulation. By R. Ottolengui, 497.

Gingival Irritations. By A. D. Black, 347.

Hydroparotitis from Artificial Teeth, 341.

Importance of Oral Hygiene. By T. P. Hyatt, 88.

Incisor Replacement, 37.

Indirect Casting Method. By C. M. Harris, 246.

Is a Child's Life Worth Six Dollars? By Dr. King, 442.

Legal Decision in the Car Case, 48.

Licensing Anesthetists, 342.

Making Artificial Stone Models. By L. Balter, 494.

Millions of Food, 341.

Mixing and Packing Amalgam, 544.

Nasal Breathing a Preventive of Caries, 86.

Natural Gum Pink, 248.

Need of Occlusal Rests, 241.

Nesbitt Removable Bridge, 40.

Oral Hygiene an Economic Factor, 41.

Oregon Supreme Court Decision, 536.

Partial Dentures. By David Baghel, 494.

Passing Strips Through Tight Contacts. By J. F. Nelson, 292.

Pathologic Conditions Under Fixed Bridges, 542.

Perforation from Drilling Canal, 398.

Perry Separator, 292.

Pepsodent, 292.

Pharmacological Action of Silver Nitrate. By Herman Prinz, 346.

Physician and His Duty to Society, 44.

Plate Polishing, 37.

Point of View, 188.

Poison Labels, 39.

Poor Impressions, 294.

Practical and Scientific Casting, 400.

Practical Hints, 540.

Practising Dentistry as a Specialty. By R. Ottolengui, 243.

Preventable Pyorrhea. By P. G. Puterbaugh, 241.

Printing on Radiographs. By J. Waters, 398.

Professor of Anesthesia. By S. G. Davis, 343.

Pulp Capping Dressing. By H. Prinz, 34.

Quick Filter, 37.

Red Cross Roll Call, 343.

Removal of Devitalized Pulps, 398.

Retaining Dentures in Hard and Flat Surfaces. By Capt. Teetzel, 294.

Rheumatic Fever from Focal Infection. By Dr. A. Lambert, 239.

Roach .Ball and Socket Attachment, 145.

Robbing the Child, 499.

Root Canal Therapeutics. By F. D. Coolidge, 398.

Root Resection. By E. H. Thomas, 293.

Rust and Steel Instruments, 42.

Saddle Bridges. By Dr. Hovastadt, 94.

Salivary Deposit Solvent. By F. W. Frahm, 347.

Sears Impression and Bite Method, 151.

Sectional Trays for Partial Impressions, 538.

Selection of Patients, 585.

Sensitiveness at the Cervical Necks, 398.

Septic Bridge-Work By Dr. Hovestadt, 96.

Short Method of Plate Polishing, 537.

Stiffening Gutta-Percha Canal Points, 443.

Strength of the Labial, Buccal Lip Functions, 581.

Stress and Strain in Bridge-Work. By A. E. Schneider, 443.

Sugar Diet and Dental Caries, 583.

Symptoms Associated with Dentition. By Dr. Ellsworth, 245.

Temporary Crowns. By H. W. Hoag, 399.

The Dental Hygienist, 97.

The First Dental School Clinic, 449.

The Radiograph Salesman. By R. Ottolengui, 87.

Training Dental Hygienists. By Anna V. Hughes, 343.

Treatment of Acute Septic Gingivitis. By A. B. Cocker, 124.

Treatment of Non-Vital Teeth, 539.

Triumph of Surgery. By Dr. Webster, 190.

Too Many Doctors, 143.

U. S. as a Going Concern, 197.

Welding Amalgam. By F. W. Frahm, 400.

What Is Being Done by the Medical Profession to Educate the Public? 446.

What Kind of Bridges Shall We Make? 581.

Why Drown? 348.

Why Not Be Sane? 540.

Why Play Golf? By Charles Evans, Jr., 247.

Why Some Gold Fillings Come Loose, 342.

Venereal Diseases and Dentistry, 45.

Vincent's Angina. By N. H. McDonald, 90.

Vulcanizing on Plaster. By E H. Mathias, 499.

### BIOGRAPHIC

Ackland, W. R., 96.

Allwine, H. W., 34.

Baghel, David, 494.

Balter, L., 494.

Barrett, Thos. J., 104.

Beach, J. W., 300.

Beals, F. L., 592.

Best, E. L., 174, 271, 317, 363.

Betts, Toplan, 82.

Black, A. D., 347.

Brown, W. H., 143.

Brush, F. C., 176.

Bosworth, H. J., 97, 585.

Bunting, R. W., 416.

Burkhart, H. J., 509.

Burnet, Jas., 365.

Calvy, P. J., 383.

Cattell, D. M., 468.

Cavanagh, W., 581.

Clapp, G. W., 425, 426.

Colyer, S., 526, 578.

Coolidge, E. D., 164, 398.

Cowling, Thos., 522.

Crocker, A. A., 100, 196.

Crocker, A. B., 145.

Curt, W. M., 41.

Crymes, F. J., 291.

Davis, Prof. S. G., 343.

Dittman, G. C., 78, 480.

Dewey, M. L., 588.

Doubleday, F. N., 454.

Ebersole, W. J., 549.

Endelman, Julio, 373.

Eller, C. A., 135.

Ellsworth, Dr., 245.

Entrinken, F. H., 392.

Evans, Charles J., 247.

Evans, George, 586.

Evans, W. A., 583.

Fink, M. A., 436.

Fischer, H. T., 239.

Frahm, F. W., 4, 53, 292, 303, 347, 375, 400, 411.

French, A. B., 182.

Gearheart, C. M., 361.

Gies, W. J., 87, 292.

Goler, G. W., 512.

Goslee, H. J., 241.

Gross, S., 68.

Gurley, J. E., 59.

Hale, J. M., 227.

Hamilton, H. B., 573.

Harris, [illegible] M., 247.

Harper, Wm. E., 13.

Hartzell, Thos. B., 24.

Head, Joseph, 99.

Homer, J., 342.

Hoag, H. W., 399.

House, M. M., 403.

Hovestadt, Dr., 94, 96.

Huet, Emile, 487.

Hughes, Anna V., 343.

Hyatt, Thaddeus, 42, 82.

Ingersol, E. S., 514.

Johnson, C. N., 395, 472, 490, 519, 542.

Junkerman, G. S., 88.

Kaiser, A. D., 517.

Karr, G. A., 181.

Kassab, A., 538.

Kells, E., 243.

King, Otto, 442.

Lambert, A., 239.

Leak, W. H., 148.

Lyons, C. J., 587.

Mathias, E. H., 499.

McCoy, J. D., 542.

McDonald, N. H., 90.

McKesson, E. J., 122.

McQuade, Jerry, 435.

Miller, A. H., 120.

Morgan, Henry W., 137, 149.

Moxham, H. C., 540.

Mummery, J. H., 386.

Nelson, J., F., 292.

Nesbitt, N. B., 539.

Null, M. M., 222.

Ottolengui, R., 86, 241, 243, 442, 497.

Peacock, C. N., 86.

Prinz, H., 195, 233, 341, 346.

Proctor, R., 449.

Puckner, W. A., 256.

Puterbaugh, P. G., 241.

Roach, F. E., 20, 37.

Rudin, N., 291.

Rattner, M., 207.

Roach, F. E., 564.

Roseberg, A. L., 446.

Rushton, Wm,. 74.

Sage, F. W., 44.

Schmitt, H. H., 537.

Schneider, A. E., 443.

Sears, V. H., 153.

Smith, B. Holly, 135, 150.

Smith, D. D., 349.

Spence, S. J., 538.

Sullivan, M. J., 549.

Teetzel, Capt., 294.

Thomas, E. H., 293.

Wallace, J. Sim, 555.

Ward, H. B., 511.

Ward, M. L., 581.

Water, J., 398.

Waugh, L. M., 98.

Webster, A. E., 190, 292.

Wendell, L., 99.

Weiss, O. A., 267.

Winslow, C. E. A., 332.

Wycoff, R. D., 253.

Younger, W. J., 547.

Zabriskie, A., 283.

ANNOUNCEMENTS

Alumni Iowa University, 489.

American Institute of Dental Teachers, 45, 591.

American Medical Association, 296.

American Society of Orthodontists, 45, 98.

Boston Session of National Dental Association, 588.

Camp Roosevelt, 592.

French-Belgian Relief Fund, 98.

Kentucky Dental Society, 98, 143, 149.

Illinois and State Societies, 98, 149.

Michigan Dental Society, 98, 149, 249, 295.

Northern Ohio Dental Association, 249.

Ohio State Society, 489.

Preparedness League, 297.

Texas State Society, 98.

U. S. Civil Service Examinations, 489.

West Virginia State Society, 98, 149.

OBITUARY

William G. Ebersole, 549.

Simeon H. Guilford, 200.

James McManus, 250.

Henry W. Morgan, 149.

D. D. Smith, 349.

B. Holly Smith, 150.

Maurice J. Sullivan, 549.

William J. Younger, 547.

BIBLIOGRAPHY

Crown, Bridge and Porcelain Work. By George Evans, 586.

Every-Day, Mouth Hygiene. By Joseph Head, 99.

Fractures and Dislocations of the Jaws. By C. J. Lyons, 587.

Modern Dentistry for the Laity. By A. A. Crocker, 100, 196.

Systematic Development of the X-Ray Plates and Films. By Lehman Wendell, 99.

### ANNOUNCEMENTS

### American Institute of Dental Teachers

The next annual meeting of the American Institute of Dental Teachers will be held at Detroit, Mich., January 27, 28 and 29, 1920. Hotel Statler will be the headquarters. A cordial invitation is extended to all persons interested in dental teaching. Russell W. Bunting, President; Abram Hoffman, Secretary, 381 Linwood Ave., Buffalo, N. Y.

### American Society of Orthodontists

The next annual meeting of the American Society of Orthodontists will be held at the Edgewater Beach Hotel, Chicago, Ill., Monday, Tuesday and Wednesday, April 5, 6 and 7, 1920. Those interested in orthodontia are invited to attend. J. V. Mershon, President, Philadelphia, Pa.; F. M. Casto, Secretary, Cleveland, Ohio.

### Campaign to Enlist the Co-operation of the Dental Profession in the Fight against Venereal Diseases by the Bureau of the Public Health Service, Washington.

The great war just ended disclosed to an astonished American people that the extensive prevalence of venereal diseases among the men drafted for military service had its source and inception principally in an infected civilian population.

To combat these diseases, Congress, when made aware of its extent and its alarming depredations into the health of the civilian population of the United States, passed the Chamberlain-Kahn Act, charging the Public Health service with the definite responsibility of organizing an effective campaign for the control of venereal

Accordingly, through its Division of Venereal Diseases, the Public Health Service outlined a plan whereby it would co-operate with state health authorities in effective venereal disease control. This provided for the appointment of an officer of each state to co-operate with the state health officer in directing the work of venereal disease control. The plan of procedure formulated was grouped under three headings, as follows:

(1) Educational measures;

(2) Medical measures;

(3) Law-enforcement measures.

The educational measures include the dissemination of information by leaflets, lectures, exhibits, moving pictures, stereopticon views, and other means, among industrial plants, commercial institutions, schools, churches, clubs, libraries, community centers, the home, and every walk of life.

The law-enforcement measures include encouragement of the closing of restricted districts; stimulating enforcement by state and municipal officials of laws and ordinances directed against prostitution in all its phases; the establishment and management of institutions for the rehabilitation of venereally infected feeble-minded persons; urging the adoption and enforcement of laws and ordinances compelling the reporting of the venereal diseases, the prohibiting of quack advertising, and the sale of venereal disease nostrums; and other measures designed to prevent the spread of venereal diseases.

The medical measures include the establishment of clinics; securing hospital facilities for venereally infected persons; making available laboratory facilities for the scientific diagnosis of venereal diseases; securing wide distribution of arsphenamin or similar products; obtaining the support of the entire medical profession by the reporting of their cases to the state boards of health, and the treating of venereally infected persons in accordance with the best modern methods; securing the cooperation of druggists in refusing to dispense venereal nostrums and directing prospective purchasers of such remedies to venereal disease clinics or reputable physicians; securing the co-operation of dentists and nurses in their respective fields of practice, and enlisting the interest and services of all medical, dental and pharmaceutical schools, societies and journals.

Under medical measures the compaign was begun with the advertising media of the country. With the end of the fiscal year June 30, 1919, out of 20,000 newspapers and magazines in the United States carrying advertising, approximately 140 only, or less than one per cent., were still carrying obnoxious advertising. In other words, more than ninety-nine per cent. are co-operating.

The next step was directed to the druggists of the country. Out of the 48,500 appealed to, the results at the end of the fiscal year showed that approximately 28,000, or nearly sixty per cent., were co-operating, and since the transfer of that campaign to state boards that number is now considerably augmented.

After the druggists, the 131,780 legally qualified physicians in the United States were appealed to, with the result that at the end of the fiscal year approximately 60,000, or nearly fifty per cent., of the medical practitioners of the country are co-operating. With the transfer of that campaign to the state boards of health this number is now also greatly increased.

Following the physicians, the campaign with the medical, dental and pharmaceutical schools for more advanced didactic and clinical teaching of the veneral diseases was undertaken, with the result that not a school in the United States has recorded an objection or entered a dissenting voice.

The campaign with the 45,000 dentists of the United States is now being launched. Before starting this campaign the Surgeon-General invited the dentists of

Washington, D. C., to a convocation held under the auspices of the National Capital Dental Society of the District of Columbia.

Following this meeting a committee of distinguished dentists in collaboration with the public health service, formulated for the campaign with the dental profession a letter, an agreement card, and an appeal similar to those used in the campaign with physicians, druggists, and advertising media.

The campaign with the dentists, therefore, consists of:

1. An "appeal" setting out every dentist's responsibility to the public health.

2. A "letter to the dentists of the United States" explaining the campaign.

3. An "agreement card" which every dentist is asked to sign and return.

Copies of these circulars may be secured of Dr. Rupert Blue, Surgeon-General, Washington, D. C.

---

### Final Decision of the Carr Case

In the United States Circuit Court of Appeals, for the Seventh Circuit. No. 2704. October term and session, A. D. 1918.

The Carr School of Preventive Dentistry and Medicine, Plaintiff-Appellant, vs. Austin F. James, Defendant-Appellee. Appeal from the District Court for the Northern District of Illinois, Eastern Division.

Before Baker, Evans and Page, Circuit Judges.

Appeal from the decree dismissing a petition charging appellee with infringing Patent No. 1,138,355, relating to dentist's tools.

Page, C. J.—Errors relied on are that the court erred in not holding:

I. That Claim 2 of the Patent in suit is valid and infringed.

II. That the infringement amounted to unfair competition.

Claim 2 is as follows: "Tools for the treatment of teeth comprising a series of straight handles, each tool adapted for use upon a predetermined shaped portion of the tooth and each having a cutting edge and a guiding portion, the guiding portion serving to engage with the tooth in advance of the cutting edge to steady the same, and the cutting edge being in the line of axis of the handle whereby the instrument has no tendency to turn when in use, and planes as contradistinguished from scraping the surface."

Stripped of all verbiage, the claim is:

First, for a guiding portion of the tool to touch the tooth in advance of the cutting edge.

Second, for a cutting edge in line of the axis of the handle.

I. The Specifications do not tell how this "guiding portion" is made or where it is or should be located. An examination of Figures 10, 11, 12, 13 and 14 in the Carr Letters Patent, conclusively indicate that the "guiding portion" is not and can not be definitely fixed anywhere but is merely the necessary contact between the side of the tooth and the side of the instrument. In Figure 14, there is no contract at all and in his testimony, patentee Carr said that in some cases, without proper adjustment of the angle of the cutting bit or blade, there could be no rest or "guiding portion."

In addition to this, the uncontradicted testimony shows that many dentists used the same sort of contact and that resting the instrument against the tooth was, in most cases, unavoidable. It further appears that in this respect there is no appreciable difference between the Carr tools and the construction of the Cravens tools and many others testified about and in evidence, made and used long before the Carr Application was filed.

II. While on first reading, the language "the cutting edge being in the line of the axis of the handle" seems simple, it will not stand analysis. The cutting edge is a line and if the language means anything, it means that that line, in construction and use, is merely an extension of the longitudinal axis of the handle.

An examination of the Carr tools shows that no tool is constructed on this plan. If any one such tool would be of use in dentistry, Carr did not make it, and one hundred and fifty such tools, as contemplated by appellant for a set, would evidently be purposeless.

If the language means that the line indicating the cutting edge is at right angles with the longitudinal axis of the handle so that the point marking the center of the line would be touched by an extension of the longitudinal axis of the handle, it will be found to be true in only one or two instances in the Carr tools, but it was and is also true in the Cravens, and numerous other tools made and used long before the alleged Carr invention.

What the language was probably intended to mean is that the center of the cutting edge is in line with the longitudinal axis of the handle. An examination of the tools in evidence shows that this is probably true generally and it also shows that in a great majority of the instruments, the cutting edge is so made that in use the tool is not pulled directly toward the operator, but must necessarily be operated by pulling or pushing against the side of the tool. If pulled directly toward you, as a Japanese plane is operated, it would neither scrape nor plane, but would merely scarify the tooth by drawing the cutting edge quartering across it. This would be a useless device.

The decree of the District Court is affirmed.

